# A Review of the National Health Insurance Scheme in Ghana: What Are the Sustainability Threats and Prospects?

**DOI:** 10.1371/journal.pone.0165151

**Published:** 2016-11-10

**Authors:** Robert Kaba Alhassan, Edward Nketiah-Amponsah, Daniel Kojo Arhinful

**Affiliations:** 1 Amsterdam Institute for Global Health and Development, University of Amsterdam, Amsterdam, The Netherlands; 2 Department of Epidemiology, Noguchi Memorial Institute for Medical Research, University of Ghana, Legon, Accra, Ghana; 3 Department of Economics, University of Ghana, Legon, Accra, Ghana; University of Glasgow, UNITED KINGDOM

## Abstract

**Background:**

The introduction of the national health insurance scheme (NHIS) in Ghana in 2003 significantly contributed to improved health services utilization and health outcomes. However, stagnating active membership, reports of poor quality health care rendered to NHIS-insured clients and cost escalations have raised concerns on the operational and financial sustainability of the scheme. This paper reviewed peer reviewed articles and grey literature on the sustainability challenges and prospects of the NHIS in Ghana.

**Methods:**

Electronic search was done for literature published between 2003–2016 on the NHIS and its sustainability in Ghana. A total of 66 publications relevant to health insurance in Ghana and other developing countries were retrieved from *Cochrane*, *PubMed*, *ScienceDirect* and *Googlescholar* for initial screening. Out of this number, 31 eligible peer reviewed articles were selected for final review based on specific relevance to the Ghanaian context.

**Results:**

Ability of the NHIS to continue its operations in Ghana is threatened financially and operationally by factors such as: cost escalation, possible political interference, inadequate technical capacity, spatial distribution of health facilities and health workers, inadequate monitoring mechanisms, broad benefits package, large exemption groups, inadequate client education, and limited community engagement. Moreover, poor quality care in NHIS-accredited health facilities potentially reduces clients’ trust in the scheme and consequently decreases (re)enrolment rates. These sustainability challenges were reviewed and discussed in this paper.

**Conclusions:**

The NHIS continues to play a critical role towards attaining universal health coverage in Ghana albeit confronted by challenges that could potentially collapse the scheme. Averting this possible predicament will largely depend on concerted efforts of key stakeholders such as health insurance managers, service providers, insurance subscribers, policy makers and political actors.

## Introduction

Universal access to good quality health care remains a major concern of health systems globally. In view of this, countries have adopted different health financing mechanisms including National Health Insurance (NHI) to ensure universal access to quality basic health care. Ghana is one of the few countries in sub-Saharan Africa spending a relatively high percentage of its Gross Domestic Product (GDP) on health. Ghana’s total expenditure on health as a percentage of its GDP was 5.4% in 2013 compared to 3.9% in Nigeria, 4.5% in Kenya, and 4.6% in Benin [1]. Likewise the percentage of government of Ghana (GoG) budget allocation to health was 10.6% of total government expenditure [2], inching closer to the Abuja target of 15% [3].

In the larger African context, a number of community-based health insurance systems have been implemented with varying outcomes. However, the operational and financial sustainability of these health insurance interventions has been a major challenge to many of these low and middle income countries in Africa, including Ghana. For instance, Kyomugisha *et al* [4] reported that the Ugandan Community Health Insurance (CHI) system was not sustainable largely because it was operated on a small budget; had low enrolment and lacked government support. Likewise, De Allegri *et al* [5] concluded that lack of clear legislation, low enrolment, insufficient risk management, weak managerial capacity and high overhead costs, are key operational challenges that threaten the sustainability of CHI in South Africa.

Sustainability of Ghana’s NHIS in this context refers to the scheme being able to operate successfully meeting its mandate without collapsing. A sustainable NHIS would mean the scheme overcoming challenges of limited funds for provider reimbursements and administrative activities; improving quality of health care and insurance services to enhance subscriber trust and increase utilization of NHIS services.

Ghana was the first sub-Saharan African country to introduce NHIS in 2003 through an Act of parliament (ACT 650, Amended Act 852) and full implementation started in 2004 (See Table 1). Under the NHIS amended Act 852 (2012), every Ghanaian is required to enroll in a health insurance scheme. This constitutional provision is however not effectively implemented because of the relatively large informal sector and weak administrative capability of the National Health Insurance Authority (NHIA) in Ghana.

**Table 1:** Overview of Ghana and its health care system.

**Socio-economic and demographic characteristics**
• Land area: 239,000sq.km
• Administrative regions: 10
• Metropolitan Municipal and District Assemblies (MMDAs): 216
• Total Ghanaian population^1^: 26.9 million (51% females & 49% males)
• Population density^1^: 109 persons per sq.km
• Rural-urban distribution^1^: 70% lives in urban/peri-urban areas & 30% lives in rural areas
• Gross Domestic Product (GDP)^2^: 7.6%
• Dependency ratio^3^: 0.72 per productive member
• Gross national income per capita (as at 2013): US$1,770
• Percentage of population working in informal sector: 67%
**Health indicators**^**4**^
• Government of Ghana (GoG) budget allocation to health (as at 2013): 10.6%
• Live expectancy at birth (as at 2013): 62 years
• Doctor: population ratio (as at 2012): 1: 10,452
• Nurse: population ratio (as at 2012): 1: 1,251
• Number of public and private health care facilities (all levels): Over 5,000
**The National Health Insurance Scheme (NHIS) in Ghana**^**5**^
• NHIS implementation started: 2004
• Legislation: parliamentary Act 650 & amended Act: 852
• **NHIA Funding sources:**
▪ National Health Insurance Levy (NHIL): approximately 70%
▪ Social security and national insurance trust (SSNIT): approximately 17%
▪ Premiums: approximately 4%
▪ Other source: approximately 8%
• **Active membership by category**
▪ Persons aged 70^+^ years: 4.5%
▪ Indigents: 4.4%
▪ Informal workers: 35.5%
▪ SSNIT contributors: 4.2%
▪ SSNIT pensioners: 0.3%
▪ Persons under 18 years: 51.2%
• **Coverage**
▪ About 40% of Ghanaians are registered with the NHIS as active members ▪ Free NHIS services to pregnant women registered with the scheme

**Source:** Information aggregated by authors based on reviewed literature

**Legend:** NHIS (National Health Insurance Authority); SNNIT (Social Security and National Insurance Trust); NHIL (National Health Insurance Levy); NHIA (National Health Insurance Authority); GoG (Government of Ghana); MMDAs (Metropolitan, Municipal and District Assemblies); GDP (Gross Domestic Product)

^1^Ghana Statistical Service (GSS). Population and Housing Census Report: Millennium Development Goals in Ghana, GSS, Accra Ghana, 2015

^2^World Development Index (WDI). Education Statistics. World Bank Data. Washington, DC, 2015

^3^World Bank. Ghana overview: recent economic developments. World Bank Official Website, 2015

^4^Ministry of Health (MoH). Wholistic Assessment of the Health Sector Programme of Work. MoH Official Website, Accra Ghana, 2014

^5^Tweneboa NA. National Health Insurance Accreditation in Ghana, Presentation, Conference Presentation. Cape Town, South Africa, 2011.

The NHIS is financed through a central National Health Insurance Fund (NHIF) which is sourced from the National Health Insurance Levy (NHIL) of 2.5% tax on selected goods and services; 2.5% of Social Security and National Insurance Trust (SSNIT) contributions, largely by formal sector workers; payment of premiums, and donor funds. Individuals who are employed in the formal sector and contribute to SSNIT are exempted from premium payment. As at 2012, over 70% of the NHIS financial inflows came from the NIL; 17.4% from SSNIT contributions and 4.5% from premium payments. Other sources of funding to the NHIF include money allocated by parliament of Ghana, grants, donations, gifts/voluntary contributions, and interests accrued from investments (see Table 1). Introduction of the NHIS became necessary after several health financing mechanisms, including Out-of-pocket Payment (OOP) failed to guarantee financial accessibility and universal health coverage to the population [6,7].

The NHIS does not attempt to treat all diseases suffered by insured members. However, many common diseases such as malaria, upper respiratory tract infections and diarrheal diseases, are covered in the NHIS benefits list. Likewise, NHIS-insured members are entitled to medical emergency care such as road traffic accidents. Also, under the free NHIS for pregnant women policy, pregnant women are exempt from NHIS premium payment. Diseases such as cancers, which are relatively not common, are not covered by the NHIS.

Overall, more than 60% of active members of the NHIS are under the premium exemption category (i.e. people under 18 years or 70+ years; pregnant women and indigents). Critics of the Ghanaian NHIS have argued that the scheme is overly generous and financially unsustainable because of the huge percentage of NHIS members under premium exemption without co-payment (subscriber sharing cost of health care with insurance provider for specified conditions) coupled with increasing cost of medical logistics/supplies and health service delivery.

According to the reviewed literature, Ghana’s policy on free NHIS for pregnant women in 2008 contributed to improved maternal health care coverage [8,9,10]. Dzapkasu et al [8], reported that free NHIS for pregnant women stimulated increased numbers of women delivering their babies in health facilities in the Brong-Ahafo region. These deliveries took place in hospitals, health centres and maternity homes. Data obtained from a health demographic surveillance system carried out in seven districts in the Brong-Ahafo region involving 91,015 women (with complete data on place of birth) showed that the proportion of babies delivered in health facilities increased from 50.1% in January, 2004 to 71.2% in December, 2009 [8].

Mensah et al [9] found in a retrospective study in Ghana (2003–2007) that NHIS-insured pregnant women (n = 140), unlike non-insured colleagues (n = 425) are more likely to receive prenatal care (85.7% against 72.0%), deliver at a hospital (75.0% against 52.9%), have their deliveries attended by trained health professionals (65.7% against 46.6%), and experience less birth complications (1.4% against 7.5%) [9]. Likewise, NHIS-insured pregnant women compared to their non-insured counterparts are more likely to benefit from postnatal check (85.7% against 70.6%) and preventive information (82.1% against 76.2%) [9]. Based on these findings, it was concluded that NHIS is an effective tool for improving health care access and health outcomes. Furthermore, grey literature statistics have shown that Institutional Maternal Mortality Ratio (IMMR) declined from 224 per 100,000 live births in 2008 to 144 per 100,000 live births in 2014. Likewise, infant mortality declined from 64 per 1000 live births in 2003 to 41 per 1000 live births in 2014 [2].

Some literature however argued that even though the NHIS contributed to increased utilization of formal health care services, the scheme did not necessarily improve the quality of health care in NHIS-accredited health facilities [11]. Quality of health care in this context includes client satisfaction with services rendered in NHIS-accredited health facilities. Proxies for health service quality include satisfaction with waiting times and queuing, availability of drugs, attitudes of staff and physical environment of health facility. The technical dimensions of quality health care include adherence to professional standards, protocols and guidelines by health care providers.

In a Ghana Health Service (GHS) annual report [11], it was indicated that introduction of the NHIS contributed to increased pressure on health infrastructure and staff. According to the GHS annual report [11], this increased pressure resulted in longer waiting times, illegal charging of fees and non-adherence to standard professional practices by health workers [11]. Patient satisfaction with the quality of health care in NHIS-accredited health facilities also remains low especially with regard to staff attitudes and long waiting times, differential treatment for NHIS-insured and non-NHIS insured clients, quality of drugs covered by the NHIS, and limited client/community engagement in NHIS activities [8,12,13].

Besides the poor quality of health care services in NHIS-accredited health facilities, some studies have suggested client dissatisfaction with NHIS district offices in terms of delayed issuance of membership cards and inadequate education for subscribers on NHIS benefits package. According to a SEND-Ghana report in 2010, some registered NHIS members did not have access to health care services in the Upper West, Northern, Upper East and Greater Accra regions because of delayed issuance of membership cards by the NHIS district offices [14]. In more recent times, delayed issuance of membership cards has improved following introduction of biometric registration system and instant card issuance by the NHIA.

Statistics on the number of Ghanaians with active private health insurance or insurance by an employer were not available to the authors at the time of this review. As at 2016, only about 40% of the total Ghanaian population (estimated 26.9 million) is enrolled with the NHIS with valid membership cards (See Table 1). The 40% enrolment in the NHIS suggests perhaps tenets of the NHIS are yet to be fully embraced by communities because of inadequate community engagement coupled with clients’ concerns on service quality in NHIS-accredited health facilities.

This review explored peer reviewed articles and grey literature on the financial and operational sustainability threats to the NHIS after a decade of its implementation in Ghana. Proposed interventions towards averting a collapse of the NHIS were also reviewed.

## Methods and Data Sources

### Criteria for eligibility and rationale

This review was based on the PRISMA guidelines for systematic reviews [15]. Conformity of this review with the PRISMA 2009 checklist has also been demonstrated in S1 Table. Literature considered for review were peer reviewed articles and grey literature on sustainability of the NHIS in Ghana. The literature search was done in the English language using keywords such as: national health insurance scheme, operational sustainability, financial sustainability, health care quality, developing countries, universal health coverage, implementation challenges, prospects, Ghana. A scoping review methodology was adopted because of inclusion of grey literature and the need to reduce reporting bias and chance effect [16]. This approach also enables the reviewers to draw sound conclusions on publications [17 cited in 16]. The review focused on the operational and financial sustainability threats and prospects of the NHIS.

### Information sources

An electronic search was done with *PubMed*, *ScienceDirect*, *GoogleScholar*, *Cochrane* and bibliographies from publications on the NHIS in Ghana. Time frame for the search was between November, 2003 and August, 2016 since the NHIS was introduced in 2003. In addition, policy documents and reports on the NHIS were obtained from databases of the Ministry of Health (MoH), Ghana Health Service (GHS), National Health Insurance Authority (NHIA) and World Health Organization (WHO) websites.

### Study selection process and exclusion criteria

The search did not include literature on health insurance in developed countries. Moreover, literature not published in English language was excluded. Articles using quantitative methodologies with sample size less than 100 were excluded because of generalizability challenges. Using the snowball approach as in Willis-Shattuck *et al* [17], the reference lists of retrieved publications were further searched which resulted in getting additional relevant publications to a saturation point where no more relevant reference lists were identified.

### Review process and literature selection

The reviewers first assessed grey literature on the NHIS to inform the development of the review background. To ensure that credible information is reviewed from the grey literature, a thorough exploration of only official websites was done to retrieve annual reports and relevant policy documents.

The next level involved screening of abstracts and full text articles in peer reviewed journals. Specific inclusion criteria used were articles explicit on: (1) NHIS sustainability threats and prospects; (2) operational sustainability of the NHIS; (3) financial sustainability of the NHIS; (4) interventions to sustain the NHIS.

All articles considered for inclusion were screened for quality by co-authors and peers who have worked substantially on the topic area. Screening for article quality was done based on rigor of methodology used in the article. Furthermore, the search process, data extraction and analysis were validated by the co-authors.

### Extraction and analysis

Data extraction was done using an Excel spreadsheet and analysis done in the narrative form since the retrieved literature is a mixture of peer reviewed articles and grey literature. This approach was used for both grey and peer reviewed literature in line with Mays *et al* [18] argument on the advantages of adopting this approach under the current circumstance.

Based on Greenhalgh *et al* [19] format, the reviewers synthesized the weaknesses and strengths of the reviewed articles in terms of quality. Moreover, content analysis of the research questions/hypotheses, methodology, sampling strategy and study findings was performed. The data synthesis enabled the reviewers to develop themes that informed a discussion on findings of the various studies.

## Results

**Fig 1:**
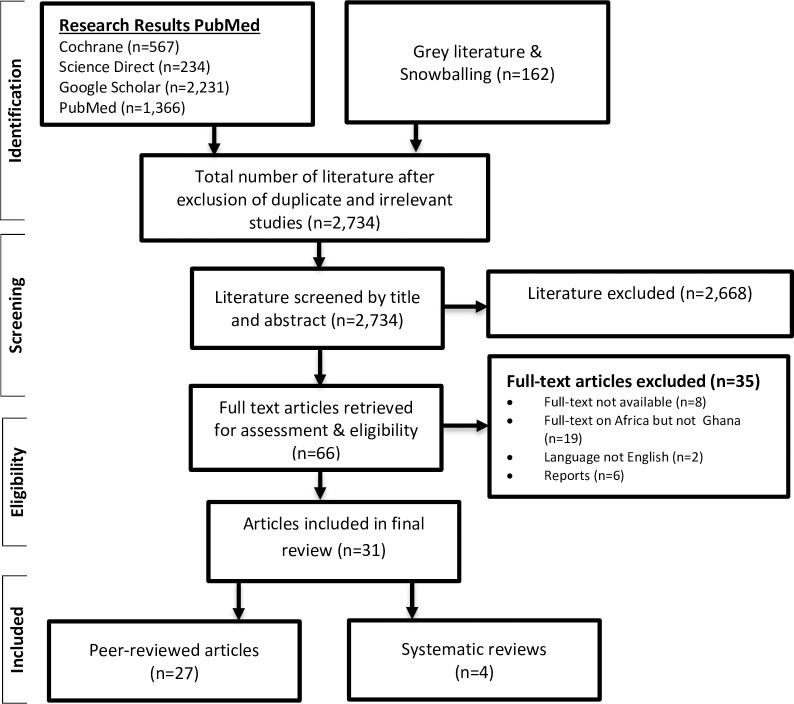
PRISMA Flow diagram of literature selection. Grey and peer reviewed articles date between 2003 and 2016.

Following the electronic search using relevant search engines, a total of 4,560 publications including grey literature were found on health insurance in Ghana and Africa. Out of the 4,560 articles, 2,734 were subsequently screened based on article title and abstracts. Next, 2,668 articles were excluded leaving 66 articles. The 66 remaining articles were further screened for eligibility in the final review and 35 articles excluded leaving 31. Finally, the 31 retained articles comprised of 27 peer reviewed articles and 4 systematic reviews. See Fig 1 below for the PRISMA flow diagram on the literature selection.

The selected 31 publications included for the final review were made up of peer reviewed articles that used primary (n = 24) and secondary (n = 7) data. Out of the seven articles using secondary data, five were narrative reviews; one was a working paper, and one was a WHO policy brief. In terms of the country context, 90% of the 31 articles were solely on Ghana while the remaining 10% were comparative studies, comparing the NHIS in Ghana with health insurance systems in Nigeria, Tanzania and South Africa.

In terms of methodology and study design, of the 24 articles that reported on primary data, 58% used quantitative approaches such as household surveys and exit interviews; 21% used only qualitative methods such as individual interviews and Focus Group Discussions (FGDs); 21% triangulated qualitative and quantitative techniques. Over 90% of the papers that used primary data also extensively reviewed relevant secondary data. The sample size of articles that used primary data ranged from a low of 28 personal interviews with six FGDs to a high of 120,000 respondents in a randomized control trial.

As shown in Table 2, 81% of the 31 reviewed articles were explicit on the sustainability challenges and prospects of the NHIS in Ghana, the remaining 19% were not. In terms of the dimensions of NHIS sustainability, 13% of the 31 articles discussed mainly financial sustainability threats; 42% were explicit on operational sustainability; 19% were not explicit on either financial or operational sustainability challenges. Eight (26%) overlapping articles discussed operational and financial sustainability challenges.

**Fig 2:**
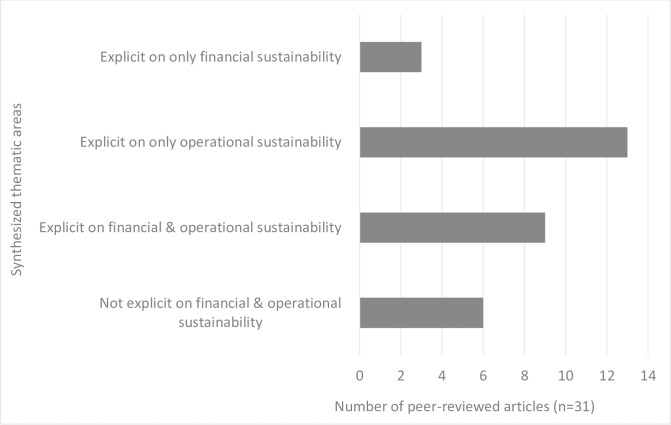
Frequency of themes in retrieved articles (n = 31). Thematic areas as presented in the Fig are the synthesized themes recurrent in the peer reviewed articles.

Proposed interventions for a sustainable NHIS in Ghana were recommended in 71% of the 31 articles; 29% were not explicit on interventions to sustain the NHIS. Out of the 31 reviewed articles, 45% recommended supply-side or provider-centered strategies; 10% suggested mainly demand-side or client-centered approaches and the remaining 16% proposed supply and demand side interventions. The thematic areas that emerged from the 31 articles for synthesis and discussion are: perspectives on threats to NHIS sustainability in Ghana, and recommendations towards sustaining the NHIS. Fig 2 shows details of the frequency of occurrence of the thematic areas discussed in the 31 reviewed publications.

**Table 2:** Perspectives on threats to NHIS sustainability in Ghana.

		Sustainability dimension (N = 31)				Proposals for sustainable NHIS (N = 31)			
Author(s)	Year	Financial	Operational	Operational & Financial	Not Explicit	Supply-side	Demand-side	Supply & Demand-side	Not Explicit
Brugiavini and Pace	2016		X			X			
Alhassan et al.	2016		X			X			
Alhassan et al.	2016		X			X			
Alhassan et al.	2015a		X					X	
Alhassan et al.	2015b		X					X	
Alhassan et al.	2015c			X		X			
Fenny et al.	2014		X			X			
Adu	2014	X					X		
Agyepong et al	2014	X				X			
Owusu-Sekyere et al	2014	X				X			
Alhassan et al.	2013		X			X			
Amporfu	2013				X				X
Odeyemi and Nixon	2013				X				X
Abiiro and McIntyre	2012			X					X
Blanchet et al.	2012				X				X
Adei et al.	2012			X				X	
Dzakpasu et al.	2012				X				X
Dalinjong and Laar	2012		X					X	
Marcha et al.	2012		X			X			
Goudge et al.	2012	X				X			
Fusheini et al.	2012			X					X
Akazili et al.	2011				X				X
Nguyen et al.	2011		X			X			
Mensah et al.	2010		X			X			
Agyepong and Nagai	2010		X				X		
Durairaj et al.	2010			X					X
Witter et al.	2009			X		X			
Ansah et al.	2009				X				X
Witter et al.	2008			X				X	
Rajkotia et al.	2007			X		X			
Baltussen et al.	2006		X				X		
**Total**	**31**	**4(13%)**	**13(42%)**	**8(26%)**	**6(19%)**	**14(45%)**	**3(10%)**	**5(16%)**	**9(29%)**

**Source:** Articles aggregated by author based on reviewed literature

**Legend:** N (Total number of peer reviewed articles)

Number of Xs in column 3 represent articles (n = 4) focused mainly on “Financial sustainability of the NHIS”

Number of Xs in column 4 represent articles (n = 13) focused mainly on “Operational sustainability of the NHIS”

Number of Xs in column 5 represent articles (n = 8) focused on both financial and operational sustainability of the NHIS

Number of Xs in column 6 represent articles (n = 6) that are “Not Explicit” on either “Financial” or “Operational” sustainability of the NHIS

Number of Xs in column 7 represent articles (n = 14) focused mainly on “Supply-side” or provider-centered interventions

Number of Xs in column 8 represent articles (n = 3) focused mainly on “Demand-side” of client-centered” interventions

Number of Xs in column 9 represent articles (n = 8) focused on both “Supply-side” and “Demand-side” interventions

Number of Xs in column 10 represent articles (n = 9) that are “Not explicit” on either “Supply-side” or “Demand-side” interventions.

### Synthesized themes in peer reviewed articles

#### Perspectives on threats to NHIS sustainability in Ghana

In all, 81% of the 31 papers included for final review reported on whether or not the NHIS in Ghana is sustainable. Fusheini et al [13] argued in their report on two local government areas in northern and southern Ghana that sustainability of the NHIS is threatened.

Fusheini *et al* [13] categorized threats to the NHIS sustainability into financial, political and operational threats. Financial sustainability threats include: fraud and corruption at health insurance schemes; abuse of the gatekeeper system where clients are supposed to first report to a primary health provider and subsequently referred to a higher level facility when necessary; low premium payments, and broad benefits package without co-payment.

Political threats according to Fusheini *et al* [13] include political interferences in the day-to-day management of the schemes and appointment of the Chief Executive Officer (CEO) by the sitting president of Ghana, which could breed political interference. Other operational sustainability threats identified in Fusheini *et al* [13] are clients moving from one health facility to another for “better” health care; delayed reimbursement of service providers; spatial distribution of accredited health facilities and staff; weak human resource capacity of NHIS district offices, and delayed production and distribution of membership cards. However, delayed production and distribution of membership cards has improved with the introduction of biometric registration and instant membership card issuance since 2013.

Another important sustainability threat discussed in the literature is poor quality of health care services in NHIS-accredited facilities. Alhassan *et al* [20–24] alluded to this challenge in their study of 64 NHIS-accredited clinics and health centers and over 1,900 households around the catchment area of these health facilities. In these studies, NHIS subscribers generally perceived they were not getting good quality health care like their counterparts paying out of pocket. Longer waiting times, poor attitudes of health staff towards clients and lack of adequate complaint avenues were highlighted as frustrating constraints for clients. Brugiavini and Pace [25] made similar observations in their studies on Ghana’s NHIS and its sustainability prospects. However, Fenny et al [26] found no significant difference in the quality of uncomplicated malaria case management of 523 NHIS-insured and non-insured patients in Ghana.

Agyepong and Nagai [27] attributed illegal modification of exemption policy under the NHIS by health service providers to delayed reimbursement of claims by the NHIA to health service providers. Duraraij *et al* [28] made similar conclusions in their review of the obstacles to the NHIS sustainability in Ghana. Ansah *et al* [29] and Witter *et al* [10] also cited higher NHIS membership versus relatively lower revenue of the NHIS, low premiums, broad benefits package and large exemption group as important challenges to the financial sustainability of the NHIS in Ghana.

As shown in Fig 2, 81% of the 31 reviewed articles discussed different types of sustainability threats to the NHIS. These threats, as reviewed in the literature, were broadly classified into financial [26,30–33] and operational threats [9,13,20–25,27,34–39]. Summary of peer reviewed articles on the NHIS sustainability challenges and prospects are presented in Table 2. Other perspectives on threats to the sustainability of the NHIS centered on equity in premium payment [38,40,41], alternate payment mechanisms [42], perceived and measured effect of health insurance on health outcomes [8,9,43].

#### Recommendations to sustain the NHIS

Recommendations towards sustaining the NHIS and enhancing its prospects in Ghana were outlined in 71% of the 31 reviewed papers. In 45% of the papers, the recommendations were supply-side (provider-centered) while 10% of the papers proposed demand-side (client-centered) recommendations; five articles (16%) proposed both supply and demand-side interventions. Sourcing for extra funding for the NHIS through oil revenue, levies on large profitable companies and increasing Value Added Tax (VAT) levy were proposed to promote financial sustainability of the NHIS [42,44].

Other recommendations made were improving revenue collection, introduction of co-payment, and capitation. Apart from the capitation system, the reviewed papers also suggested the adoption of cost control mechanisms such as enforcement of the gatekeeper system; implementation of stringent monitoring mechanisms on health providers, and improving referral systems [28,44]. The need for policy reforms on the low premiums and generous broad benefits package under the current NHIS were also explored as possible interventions to financially sustain the NHIS [28,45].

Other proposed interventions to ensure operational sustainability of the NHIS include: improving geographical accessibility of accredited health facilities through infrastructural expansion; improving material and human resource capacity to reduce workloads in health facilities [13,34]. Some of the reviewed papers recommended improvement of staff motivation levels; early reimbursement of health providers, and human resource capacity development at NHIA district offices [13,23,24,27,28] as means to sustain operations of the NHIS. Community education, participation and more autonomy of district NHIS offices were discussed as critical intervention areas towards sustaining Ghana’s NHIS [36].

Finally, suggestions were made on the need to de-couple politics from the routine management activities of the NHIS since political interferences could stifle the progress and sustainability of the NHIS [13,28]. Instances of political interference in recruitments and appointment of NHIS employees were alluded to by Fusheini *et al* [13].

## Discussion

This review explored empirical and grey literature on sustainability of the NHIS in Ghana. Overall, the reviewed literature reported emerging challenges of the NHIS such as administrative constraints, poor quality of health care to NHIS-insured clients, political interference and limited revenue sources. These challenges were highlighted as potential sustainability threats to the NHIS.

Different perspectives on the NHIS sustainability were synthesized into two broad categories: namely operational and financial sustainability. Operational sustainability was discussed in 42% of the reviewed papers. Financial sustainability was discussed in 10% of the papers (see Table 2). This trend is consistent with recent reports on sustainability of the NHIS by SEND-Ghana [14] and the NHIS annual report [46].

Even though concerns have emerged on sustainability of the existing funding sources for the NHIS in Ghana [13,28,33,37,42,44,45], a significant number of the reviewed literature [7,9,34–36,38,47] in this paper argued that managerial and administrative challenges such as quality of health care rendered in NHIS-accredited facilities pose significant viability challenge to the NHIS than funding since the scheme is predominantly tax-funded.

Another observation in this review is the percentage of papers (71%) that were explicit on proposed interventions to sustain the NHIS. This percentage suggests that there is sufficient scientific information available to policy makers and stakeholders of the NHIS to inform policy discussions on sustainability strategies for the NHIS. Relying on scientific data to inform policy decisions is a better alternative to anecdotal information [48].

It was however observed that, many reviewed papers were not focused on demand-side (client-centered) sustainability strategies. Only 10% of the 31 reviewed papers (see Table 2) carried clear messages on adopting client-centered approaches (i.e. emphasis on customer care and client satisfaction) to promote sustainability of the NHIS in Ghana.

Client-centered interventions such as community participation in health have proved successful in mobile phone technology for health (MOTECH), and other *m-Health* initiatives in Rwanda [49], Nigeria [50] and Ghana [51,52]. Active involvement of users of health care and health insurance services could therefore be an important intervention and new policy direction to improve quality health care and ensure accountability to clients by health providers and health insurance authorities.

It is recommended that, more prevalent scientific studies and reviews be conducted on client-centered quality services at various stages of implementation of the NHIS. Since there is growing recognition of the operational challenges confronting the NHIS in Ghana, adopting client-centered quality health care and health insurance services could enhance perceptions on the NHIS and increase client trust in the scheme.

Key impediments towards attaining client-centered health care and health insurance services in Ghana include geographical inaccessibility to health facilities, limited information on benefits package, undue delays in accessing health services, perceived differential treatment for NHIS-insured and non-insured clients, informal fee charges, delayed issuance of membership cards and limited complaint avenues for aggrieved clients. These limitations have been discussed in detailed in the reviewed articles [10,27,34,36,38,44].

In response to many of these challenges confronting the NHIS, the NHIA initiated the instant issuance of membership cards, biometric registration of members and establishment of Client Call Center to handle grievances of NHIS subscribers. Even though there are inadequate scientific impact evaluation studies on the effect of these interventions on quality service provision and decisions to (re)enroll in the NHIS, these interventions have improved client and health staff perceptions on the NHIS in some cases [20,23,24].

It is also important to emphasize the need to complement client-centered services with efforts to satisfy health care providers in the areas of early reimbursements, infrastructural improvement, improving staff numerical strengths and motivation levels, availability of drugs and capacity building for health workers. These areas have been cited as important supply-side interventions that could help ensure active stakeholder participation in the NHIS and curtail unprofessional conduct of health providers [27].

Furthermore, to attain client-centered quality services at the levels of the NHIS and the health care providers, there is the need for strict monitoring and supervision by the national, regional and district NHIA offices. This will not only help identify genuine operational challenges but also detect illegal acts such as illegal charging of fees and other administrative lapses.

All in all, the reviewed literature demonstrated that only few papers discussed client-centered interventions towards sustaining Ghana’s NHIS. In addition, even though many papers used triangulated methodologies, the emphasis was on quantitative data. Albeit large sample sizes were used in many articles, one common weakness was the lack of nationally representative sample sizes. Moreover, none of the studies did a detailed analysis of databases that explored perspectives of the three key stakeholders of the NHIS: client, provider and insurer. Discussions on the roles of the provider, client and insurer towards sustaining the NHIS were largely done in isolation, thus creating a gap that needs to be bridged in future scientific endeavors.

Even though many articles examined operational sustainability of the NHIS, not much was discussed on the role of clients and communities in sustaining health insurance operations. This suggests the need to intensify efforts towards involving clients and community members in quality service monitoring and improvement. Existing platforms such as the Client Call Center instituted by the NHIA presents an opportunity to effectively involve clients in health insurance planning and implementation.

Community engagement in NHIS activities is key to promoting client interest and compliance to health insurance policies. Studies by Alhassan et al [23,24] found that engaging existing community groups and associations in monitoring health and insurance service quality could help improve service providers’ accountability to clients, health worker motivation levels and relationship with clients. Moreover, community engagement in health has been found to enhance client adherence to medication regimen and honoring of clinic appointment dates [24]. These findings suggest, community involvement in NHIS activities will not only create awareness on new policies but also promote stakeholder support and goodwill for the insurance scheme.

### Limitations

The current review is limited by the type of literature retrieved for review. First, the literature sources were all searched in the English language. It is possible relevant literature published in other languages were left out to the detriment of quality results and discussions.

Secondly, due to limited relevant information in the topic area, grey literature was reviewed to complement findings of the peer reviewed articles. This might have inadvertently introduced reporting bias by the authors. Even though quality checks were done through credible websites and validation of findings by expert reviewers, reported information on grey literature could still be bias.

Finally, the review is largely limited to the NHIS in Ghana and would be most appropriate in discussions pertaining to the Ghanaian context. Nonetheless, lessons could be drawn from the peculiar case of Ghana by other African countries which are at various stages of health insurance implementation. Future reviewers should extent their scope and do comparative reviews of health insurance sustainability in more than one African country.

## Conclusions

This paper reviewed relevant literature on sustainability challenges of the NHIS and suggested interventions to avert a collapse of the scheme. Reviewed articles generally acknowledged some sustainability threats confronting the scheme. Many of these articles discussed the administrative/operational sustainability threats while a few discussed the financial sustainability threats.

Discussions in this paper are not exhaustive, instead the debate has been initiated on the need to prioritize client-centered quality service approach (i.e. customer care and client satisfaction strategies) at the health insurer and service provider levels. It is expected that complementing existing efforts with these strategies will contribute towards increasing confidence in the NHIS to promote membership participation and retention.

## Supporting Information

S1 Table PRISMA 2009 Checklist. (DOC)
